# Some Medical Hæmorrhages

**Published:** 1907-12-14

**Authors:** Leonard Williams

**Affiliations:** Physician to the French Hospital, Assistant Physician to the Metropolitan Hospital.


### Hospital Clinics.

### SOME MEDICAL HÆMORRHAGES.

By LEONARD WILLIAMS, M.D., M.R.C.P., Physician to the French Hospital, Assistant Physician to the Metropolitan Hospital.

A paper read before the Æsculapian Society.

If we would understand the clinical significance of any particular hæmorrhage, it is essential that we should have clear ideas on the subject of hæmorrhage in general. I make no apology therefore for attempting to approach my subject from a panoramic rather than from a microscopical standpoint.

By hæmorrhage is usually understood the passage of blood out of its normal channels either into the outer air or into tissues which receive it reluctantly, and immediately set to work to dispose of it. Hæmorrhage is therefore obviously due to a breach of continuity in a blood-vessel. The most obvious cause of such an accident is external violence, which, though it may give rise to such conditions as cerebral, pancreatic, and other internal hæmorrhages, does not come within the scope of my present subject. The other methods by which loss of blood may occur are closely connected with the important subject of blood-pressure. It may occur by the normal pressure acting on inadequate or diseased vessels, or it may result from a supernormal pressure acting on vessels which are virtually and clinically normal. It is, of course, when both these abnormalities are present simultaneously—when, that is, we have a supernormal pressure acting upon diseased or degenerated vessels, that the conditions are most favourable for the occurrence of hæmorrhage. I do not lose sight of the fact that poisons, whether they be organic or inorganic, are very liable to give rise to hæmorrhages; but inasmuch as the means by which they act are still hidden from us, we are entitled to assume that these means are well within the above category.

Instances of the result of the action of a normal pressure upon inadequate or diseased vessels are to be found in hæmophilia, hæmorrhagia neonatorum, carcinoma, gastric ulcer, and the like. Instances of the action of supernormal blood-pressure upon vessels which are to all intents normal may be seen in some forms of epistaxis and retinal hæmorrhage, in mitral stenosis, in gastrotaxis, and similar conditions. Examples of a supernormal blood-pressure acting on diseased or degenerated vessels are easy to recall; of such are all cases of ruptured aneurysms, most cases of apoplexy, the hæmatemesis of hepatic cirrhosis, and the hæmaturia of granular kidney. If, then, we dismiss the action of a normal blood-pressure upon inadequate or diseased vessels, as being in part too obvious and in part too intricate for our present purposes, we are left with those causes in which a supernormal blood-pressure takes a leading part.

Now, what is the clinical significance of supernormal blood-pressure, or, as it is the unfortunate fashion to call it, high arterial tension? We know what it may lead to; but how does it arise, and how are we to behave in its presence? High blood-pressure of a transient nature may arise in a variety of ways, but the sustained high blood-pressure with which we are now concerned arises mainly, if not exclusively, in two ways. It may be the result of toxins circulating in the blood, which either directly or by way of the nervous system constrict the peripheral vessels; or it may be the result of a purely defensive measure on the part of the organism, induced voluntarily and of malice aforethought, as necessary to the continuation of life. The attitude which we should adopt in its presence must therefore be dictated solely by the causes which have produced it. If we fail to interfere in the first case we are little better than ignorant accessories before the fact; if we presume to interfere in the second, we shall probably have to answer to a very serious charge preferred by our own consciences, even if we escape the attentions of some less accommodating tribunal. Let us then consider some instances of these two causes, and let me ask your forgiveness if for the sake of brevity I seem unduly to dogmatise in matters, the understanding of which is still darkened by the ink of controversy.

It is not, I think, necessary to define the use of the word toxins in this connection beyond saying that it is a generic term used to cover certain products of disordered or defective metabolism whose composition is little if at all understood, but whose effect is that of causing a general vaso-constriction. These toxins are believed on sufficient grounds to be manufactured in the gastro-intestinal canal, and their presence is unquestionably associated with what is known as the gouty diathesis; the chronic morbid condition, namely, which is liable to dog the footsteps of those who eat too much meat, who breathe too little oxygen, and take too little exercise. By direct irritation of the walls of the vessels, or by irritation of the vaso-motor centre in the medulla, these toxins cause the peripheral vessels to contract, and the blood-pressure immediately rises. The degree of this increment depends, of course, upon the degree of the toxæmia and the length of time during which it has been in operation. In a case of moderate severity, in which the manometer, had it been consulted, would during a period, say, of a few months, have registered 160-180 mm. Hg, there may be no symptoms sufficiently definite to bring the patient to his doctor. He may perhaps notice an undue amount of breathlessness on slight exertion, or momentary attacks of slight giddiness: more often he will notice nothing, though his friends will observe that he has seemed lethargic and out of sorts, and is becoming increasingly irritable. These things are not at all unusual in a busy man who is nearing 50 years of age, and so it is generally agreed that he is overworked and is in need of a tonic and a holiday. His wife's maiden sister therefore gives him some pills containing iron and strychnine which did her so much good when she was run down. And these he takes. Consequently, one evening after dinner, he is stooping down to pick up a card which has fallen on to the floor, and his nose begins to bleed. The bleeding is not very profuse, but it persists in spite of the cold bunch of keys which is applied to his neck; so the doctor is sent for. Now, a most searching examination of this man's condition by the ordinary methods will fail to reveal anything seriously disquieting. His heart is not enlarged; there are no murmurs; the sounds, indeed, are unusually clear and sharp, especially that at the aortic base; the beats are strong and regular. The urine is free from albumen and sugar, and its specific gravity is normal. Are we, then, to conclude that the bleeding is of no consequence, and that a pledget of wool soaked in adrenalin and applied to the bleeding point, combined with a night's rest, is all that is necessary to restore the patient to health? We must conclude nothing of the sort. The accentuation of the second sound at the aortic base ought at once to arouse our suspicions, and we should immediately proceed to estimate the blood-pressure. If no manometer is at hand, we can at least count the pulse in the upright and recumbent postures. We know that, normally, the rate lying down is slower by ten to twelve beats per minute than the rate standing up; and we know further that if this difference is abolished, the arteries are unduly contracted. In such a case as I am supposing, that is exactly what we should find, and the manometer would certainly register from 180 to 200 mm. Hg, or even more.

Such a man is on the brink of a precipice. He has had what the French call an "epistaxis de la quarantaine" (an epistaxis of 40 years of age—they are more precocious over there!), and if we fail to read his symptoms aright we allow him to drift blindly, and even joyously, to his inevitable doom. For the next hæmorrhage, remember, may not be from his nose: it may be into his eye, to blind him; or into his internal capsule, to cripple, perchance to kill, him. His immediate necessities can be met by rest, warmth, and vigorous mercurial purgation, and it would be right to administer nitrite of amyl, erythrol tetra-nitrate, or other vaso-dilators; but the best thing of all, if we have but the courage, is to bleed him. But whichever course we adopt, unless we take steps to guide him into the paths of physiological rectitude, his respite will be brief. He may not, indeed, suffer another hæmorrhage; but his arteries, which are now but functionally contracted, will, by reason of the constant strain upon them, soon become organically implicated and undergo sclerotic and other degenerative changes. To these changes various names are applied. In the larger vessels we call it atheroma, the mother of aneurysm and angina pectoris; in the smaller vessels we call it arterio-sclerosis, the results of which vary with the site of its selection, for of all the morbid processes this is surely the most capricious. An arterio-sclerosis of the kidney is called chronic interstitial nephritis, an arterio-sclerosis of the liver is dubbed cirrhosis; but no matter by what name we call it, let us above all things remember that the essential condition is a degeneration of the arterioles, which degeneration has been provoked by the long-continued strain of a high blood-pressure which was originally functional and curable. Now, how are we to guide him so that he may avoid these horrors? How shall we so direct him that his blood-pressure, which now threatens him, shall be permanently reduced to reasonable proportions? The answer to such questions involves matters of detail into which it is impossible here to enter, but I may be permitted to record my emphatic and ever-deepening belief that purely vaso-dilative drugs are of little avail, and that the best, if not the only means, to this end are substantially comprised in King Henry's advice to Falstaff: "Fast, forswear sack, and live cleanly."

In connection with the foregoing we must bear in mind that epistaxis is by no means the only symptomatic hæmorrhage which may overtake a person who is suffering from high blood-pressure. Such a person may, on the contrary, bleed from anywhere. He may have hæmoptysis, hæmatemesis, hæmaturia, melæna, and other forms of hæmorrhage; the site is determined by the point of least resistance. What we have to remember is that a slight oozing, whether it be from the nose or elsewhere, which is superficially trivial, may reveal to the seeing eye a state of matters which, if allowed to go unremedied, is fraught with the gravest consequences.

Let us now look at epistaxis from the other point of view. You are asked to see a roseate, rather bulky woman of thirty, who has had a fairly profuse attack of bleeding from the nose which refuses to yield to ordinary household remedies. Your inquiries elicit that it came on suddenly after she had been scolding one of the children; that she certainly had been increasingly breathless of late, but had been otherwise in her usual health. The pulse is regular and presents no very suggestive characters. The case of the man whom we have just been considering, is fresh in your memory, and you proceed to take this patient's blood-pressure. The manometer registers 170 mm. Hg—a high reading. Are, then, the two cases identical in kind, or at any rate very similar? In both we have full-blooded people in whom dyspnœa, the dyspnœa of slight effort, has been the only noticeable symptom; in both, there may have been lethargy and irritability of temper; in both, the hæmorrhage has followed suddenly on some event of everyday occurrence; in both, the manometer shows a reading which is definitely supernormal. Are we, then, to treat the woman as we have treated the man; are we to give her nitrite of amyl and warn her of the possibility of a cerebral hæmorrhage; are we to assure her of her organic soundness, and urge her to take plenty of exercise? We must do none of these things until we have completed our examination. So far, we have not examined the heart, and until we have done so, we are not entitled to give any opinion upon any matter which relates, however remotely, to the circulation. In the case of the man, you will remember, we found this organ normal as to size: the sounds pure, with the second sound at the aortic base unduly accentuated. In the case of the woman, an examination of the cardiac area at once reveals the cause of her epistaxis, for she has the enlarged right ventricle, the rough presystolic murmur, and the accentuated second sound at the pulmonary base which speak so loudly of a constriction at the mitral opening. Now, a mitral stenosis indicates venous engorgement; the veins are unable to pass their blood onwards, and a small vessel in an unsupported situation, say in the mucosa of the nose, bursts, and a hæmorrhage, an epistaxis, ensues. That is simple enough, but how comes it that the manometer shows so high a reading in a disease the essence of which is that the veins should be engorged and the arteries comparatively depleted? Is it that the instrument is useless, or is it but another example of the paradoxes with which Nature delights to puzzle those who attempt to read her secrets? It is the latter rather than the former, and yet it is very simple. Arterial blood is essential to all the cells in the body, but more especially is it essential to the medulla. It follows, therefore, that the arterial pressure must always be higher than the venous. If for any reason the pressure in the veins becomes raised, as in mitral stenosis it necessarily does, the pressure in the arteries must also become raised to a corresponding degree. If not, the medulla is deprived of arterial blood, and the patient dies at once. This is the phenomenon to which I referred when, at the outset, I said that high blood-pressure might be, and often was, a purely defensive measure on the part of the organism.

In the light of these considerations it is not very difficult to see wherein our two cases of epistaxis, that of the man and that of the woman, though bearing a strong superficial resemblance, differ fundamentally in their diagnosis, prognosis, and treatment. The prognosis in the case of the man is good; that in the case of the woman is at best sombre. The treatment in the one case is to reduce arterial tension and relieve the strain on the left ventricle. The treatment in the other case is to reduce venous tension and relieve the strain upon the right ventricle. I would not be so presumptuous as to discuss with you the measures proper to the management of an ordinary mitral stenosis, though in these progressive days our ideas even upon so well-worn a subject are undergoing considerable modification, but I should like to add a word to what I have already said about the man's case—the case of high arterial tension due to vaso-constrictor poisons circulating in the blood. Such cases are frequently, far too frequently, diagnosed as granular kidney. The mistake is pardonable, but it argues a lack of judgment. It is the more pardonable inasmuch as many of these functional cases how not only a perceptible measure of ventricular enlargement, but a degree of albuminuria which is quite unmistakable. Were I not afraid of wearying you, I could give the details of several cases which had been positively pronounced to be chronic interstitial nephritis by very competent and painstaking physicians, which time and the results of treatment proved to have been purely functional. The moral of this is two-fold. In the first place, high arterial tension, even when accompanied by albuminuria and slight cardiac enlargement, is by no means necessarily due to organic disease either in the kidneys or elsewhere; and in the second, that granular kidneys, hepatic cirrhosis, and general arterio-sclerosis are almost invariably preceded by, if indeed they are not directly due to, a state of functional high tension, which is eminently curable. The detection of this functional stage is not always easy, but if we avail ourselves of instruments of precision in estimating blood-pressures, and are alive to the spectres which may lurk behind such apparently trivial matters as a slight epistaxis, we shall often be rewarded with the satisfactory sensation of having surely averted a grave disaster.

Hæmoptysis is an accident of very frequent occurrence. It is commonly assigned to two main causes, namely, phthisis and mitral stenosis. As it has recently become the fashion to treat this condition, however arising, by the administration of nitrite of amyl, it may not prove altogether unprofitable if we consider the matter for a few moments. Nitrite of amyl, as you know, is a powerful vaso-dilator, prompt and certain in its action, though transient in its effects. The consequence, therefore, of causing its inhalation is to induce a widespread dilatation of the systemic vessels, with a considerable and rapid fall of arterial pressure, but no necessary, or even probable, consequential effect on the vessels in the pulmonary system. Hæmoptysis may mean the rupture of a vessel in the pulmonary system proper, or it may mean the rupture of a vessel in the systemic system, for the lungs are supplied by the bronchial arteries which are branches of the thoracic aorta. Now, if the ruptured vessel be in the pulmonary system, then nitrite of amyl, though it may do no harm, will certainly do no good. Where the hæmoptysis is due to mitral stenosis, it is always a pulmonic vessel which is affected, so that in this condition, at any rate, nitrite of amyl is useless. The hæmoptysis of phthisis may mean the rupture either of a pulmonic or a systemic vessel—the excavating process treats them with brutal impartiality—so that the utility of the nitrite depends upon which is affected. When it is a pulmonary vessel, we know that the drug is useless when it is a systemic, however, we may be certain that withdrawing the blood into other channels will arrest the hæmorrhage. The question which we have to ask ourselves, is whether in employing this drug in this condition we are running any risks. The answer is that a certain measure of risk must be undertaken in most therapeutic endeavours, more especially in emergencies, and that the best way of avoiding them is to realise their existence. In this case the risk certainly exists, and may be briefly stated as follows. In tuberculosis generally, and in advanced tuberculosis always, the mean arterial pressure is low—so low that the medulla finds some difficulty in maintaining its supplies. A hæmorrhage still further reduces this pressure. If now, with a view of checking the hæmorrhage, we bring about a widespread vaso-dilatation, the pressure is still further reduced, and is consequently in serious danger of being depressed below the level at which life can be sustained. I do not want to disparage the treatment of tuberculous hæmoptysis by nitrite of amyl (I have seen it act like a charm), but I submit that we do well to realise its limitations, and to bear in mind the fact that its employment may conceivably precipitate the event which we are anxiously trying to avert.

There is, however, a form of hæmoptysis in which nitrite of amyl can do nothing but good. We have seen that a man who has high blood-pressure due to toxæmia may bleed from anywhere. The site of the hæmorrhage is merely a question of his *locus*

*minoris resistentiæ*; and if that happens to be in a branch of one of his bronchial arteries instead of in his nose, he will have a hæmoptysis instead of an epistaxis, and whether it be the former or the latter, the hæmorrhage is properly and effectually treated by nitrite of amyl. Hæmoptysis due to high arterial tension is an event of much more common occurrence than is generally supposed, and I am inclined to believe that of the many recorded cases of hæmorrhage from the lung which have been successfully treated by this means, some at least have been cases of high arterial tension mistakenly labelled phthisis. Here is an instance. A man of fifty was admitted into hospital with hæmoptysis, and the senior resident applied nitrite of amyl to his nostrils, whereupon the hæmorrhage ceased. I saw him the same afternoon; he did not look to me like a tuberculous subject, but for fear of re-inducing his hæmorrhage I refrained from examining his lungs, contenting myself with a sufficient examination of his præcordial area to be sure that he was not suffering from mitral stenosis. On the occasion of my next visit I asked the resident if he had made out the cause of the hæmoptysis, and he replied that the patient was tuberculous and displayed definite physical signs at one apex. I made a very careful examination, but was unable to confirm this view. Such physical signs as were present—and they were not very marked—were quite compatible with the disturbance which a hæmorrhage necessarily causes to the portion of lung from which the blood has issued; and there was nothing in the history or the patient's condition to support the theory of tubercle. On examining his heart with more care than I had previously done I found that his second aortic sound was much increased, and on testing his blood-pressure I found it to be 200 mm. Hg, and that, in spite of the fact that he had been carefully treated for three days. This finding at once excluded phthisis, and showed plainly that the hæmoptysis was caused by a supernormal blood-pressure, which happily proved to be functional.

There is one other form of hæmorrhage which seems to demand more than a passing notice—namely, cerebral hæmorrhage. Apoplexy we know to be brought about in most instances by a high blood-pressure acting upon arteries which are already degenerated. When the rupture takes place, the effused blood acts the part of a foreign body, and the intracranial pressure is at once raised. Now, this augmentation of the intracranial pressure is a very important fact to remember; for the augmentation renders it much more difficult for the arterial blood to penetrate inside the skull-cap, which it must do if the medulla is to be supplied and the patient kept alive. Let us now suppose that we have a patient who has had what is undoubtedly a cerebral hæmorrhage. You are expected to do something; you feel that you must do something. What are you to do? The first thing to do is to place the patient on his side, preferably the paralysed side, so that his breathing may not be obstructed by the relaxed and paralysed structures in his air-passages. You apply an ice-bag to the head; you see that the rectum is washed out, that the bladder is relieved; and you give such instructions as will ensure, as far as possible, the prevention of bedsores. Now comes the important question, Ought you to bleed him? The object of venesection in such a case is to lower blood-pressure so that the hæmorrhage may not continue. That is a very desirable end to attain, because, if the hæmorrhage does continue, the lateral ventricles will soon fill with blood and the patient will die. But, important as this end undoubtedly is, we must on no account allow it to obscure our sense of perspective. Inside that cranial cavity there is not only a ruptured vessel, whose bleeding we wish to control, there is also a structure called medulla oblongata whose supply of arterial blood must at all hazards be maintained. If, then, we reduce blood-pressure to the point at which the reduction will be effective in checking the hæmorrhage, we are obviously in danger of reducing it to the point at which the medulla is starved. There may be a margin of safety, a point to which you may reduce the blood-pressure so as to moderate the hæmorrhage, without seriously diminishing the supplies to the medulla; but surely this is a razor's edge on which no practical physician will voluntarily choose to tread. The manometer has no information to give us on this crucial point. It tells us, no doubt, that the arterial pressure is very high, but we know that the arterial pressure was high before the accident, and that it is now higher still because it has to overcome an augmented intracranial pressure; but the instrument does not, and cannot, tell us whether we ought to bleed the patient at all, and, if so, what are the danger signals. For there are no danger signals. When the arterial pressure is reduced below the intracranial, death is instantaneous. That venesection may be resorted to in apoplexy not only with impunity, but with conspicuous benefit, is a fact which must be accepted on the testimony of very competent physicians; that it is at best a dangerous expedient, dangerous to the life of the patient and extremely dangerous to the reputation of the practitioner, the above considerations are surely sufficient to show.

I do not mean to be understood that the manometer is a useless instrument where apoplexy is concerned. It is, on the contrary, of the very highest utility. In the first place it will help to guide us as to whether it be indeed a hæmorrhage, and not a thrombosis, which has produced the symptoms. Theoretically, the diagnosis between these two conditions is easy; practically, it presents considerable difficulties, to the solution of which the use of the manometer will often assist us; for whereas in hæmorrhage the blood-pressure is invariably high, in thrombosis it may be, though it is not necessarily, normal or even subnormal. The instrument will also help us in the very difficult task of forming a prognosis; for if the blood-pressure does not rise after the attack, especially if there is no progressive rise, then we can afford to be of good cheer. If, however, the blood-pressure shows an increase each time it is tested, then it means that the hæmorrhage is continuing in spite of the obstacle of the increased intracranial pressure, that the blood is ploughing its inexorable way into the lateral ventricles, and that the unequal combat will shortly be succeeded by a disastrous and perpetual peace.

